# Supplementary material for: Remote epitaxy of single-crystal rhombohedral WS2 bilayers
Source: Nat Commun. 2024 May 16;15:4130. doi: 10.1038/s41467-024-48522-8 (PMC11099013; doi:10.1038/s41467-024-48522-8)
Supplement: Supplementary file 1 — Supplementary Information [file 41467_2024_48522_MOESM1_ESM.pdf]

Supplementary information for

Remote epitaxy of single-crystal rhombohedral WS_2 bilayers

Chao Chang[#], Xiaowen Zhang[#], Weixuan Li[#], Quanlin Guo[#], Zuo Feng[#], Chen Huang, Yunlong Ren, Yingying Cai, Xu Zhou, Jinhuan Wang, Zhilie Tang, Feng Ding, Wenya Wei*, Kaihui Liu*, and Xiaozhi Xu*

The supplementary information includes:

Supplementary Note 1

Supplementary Fig. 1-16

Supplementary Note 1: Modelling Details.

The free energy of the bilayer WS_2 is mathematically expressed as

$$E_f = 3L_1 E_{edge_1} + 3L_2 E_{edge_2} - S_1 E_{vdW_1} - S_2 E_{vdW_2}, \quad \text{S1}$$

where L_1 and L_2 are the edge length of the first layer and second layer WS_2, respectively. E_{edge_1} and E_{edge_2} are the edge formation energy of the first layer WS_2 on substrate and second layer WS_2 on the first layer WS_2, respectively. E_{vdW_1} is the van der Waals interaction between substrate and WS_2, E_{vdW_2} is the van der Waals interaction between WS_2 and WS_2.

The edge energy of first layer WS_2 was calculated based on a series of triangle models with identical edges on substrate. Considering S source is usually excess in the growth of TMDs, we chose the 100% S-passivated W zigzag edges due to its high stability at an S-rich environment. Overall, the edge energy is calculated by

$$E_{edge_1} = (E_{L\text{-}6} - E_{L\text{-}5} - \Delta E_{vdW} - \Delta n_{WS_2} * \mu_{WS_2} - 4 * \mu_S)/3(L_6 - L_5), \quad \text{S2}$$

where $E_{L\text{-}6}$ and $E_{L\text{-}5}$ are the total energies of two WS_2-substrate systems with different length of WS_2 triangles (6 unit cell and 5 cell unit, respectively), ΔE_{vdW} is the vdW energy difference between substrate and different length of WS_2 triangles. Δn_{WS_2} are the different numbers of WS_2 units between two WS_2-substrate systems; μ_{WS_2} is the energy of per unit of WS_2. μ_S is the chemical potential of S calculated from S_8 molecule to represent a S-rich environment.

The Gibbs free energy of bilayer WS_2 is calculated to estimate the nucleation barrier by

$$G = E_f - (\Delta u_{w_{source}} + 2\Delta\mu_S - \Delta\mu_{WS_2}) * n_{WS_2}, \quad \text{S3}$$

where $\Delta\mu_{w_{source}}$ is the chemical potential difference of W source under different partial pressure of W and temperature. $\Delta\mu_S$ and $\Delta\mu_{WS_2}$ are the chemical potential difference of S source and WS_2, which are invariable due to the usually excess S rich environment and invariable pressure/temperature in the

growth chamber.

$\Delta\mu_{w_{source}}$ is calculated by

$$\Delta\mu_{w_{source}} = h_{w_{source}}(T, P_0) - h_{w_{source}}(0, P_0) - TS(T, P_0) + k_B T ln(\frac{P_{w_{source}}}{P_0}). \qquad \text{S4}$$

Based on the classical nucleation theory, the 2D nucleation rate is defined as

$$R_{nul} = R_0 \exp - (G^*/k_B T), \qquad \text{S5}$$

where R_0 is the pre-factor for WS_2 nucleation, G^* is the nucleation barrier, k_B is the Boltzmann constant and T is the temperature.

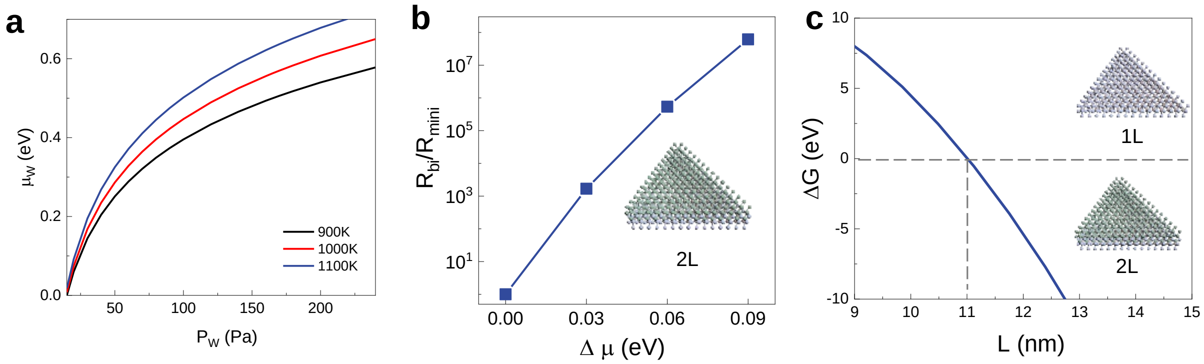

Supplementary Fig. 1 | Mechanism of the uniform nucleation and growth of bilayer WS_2. a, The $\Delta\mu_W$ versus the partial pressure of W source at different temperature. **b,** The nucleation rate of bilayer WS_2 with different $\Delta\mu_W$, where $\Delta\mu_W$ is the chemical potential difference of the W. **c,** The Gibbs energy difference between bilayer and monolayer WS_2 on *a*-plane sapphire. Bilayer WS_2 is more thermodynamically favourable than the monolayer one beyond a small critical size (~11 nm).

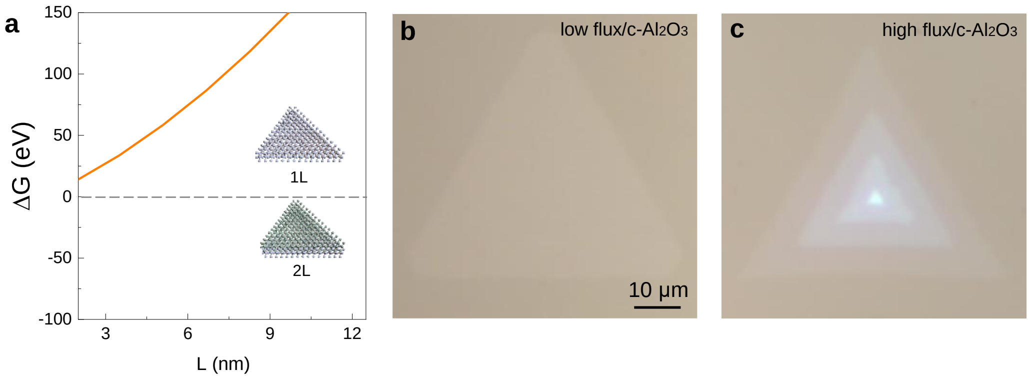

Supplementary Fig. 2 | Growth of WS_2 on *c*-plane sapphire. a, The Gibbs energy difference between bilayer and monolayer WS_2 on *c*-plane sapphire. Monolayer WS_2 is much more thermodynamically favourable than the bilayer one. **b-c,** Optical images of WS_2 islands grown with low **(b)** and high W flux **(c)**. The image sizes of **(b)** and **(c)** are the same.

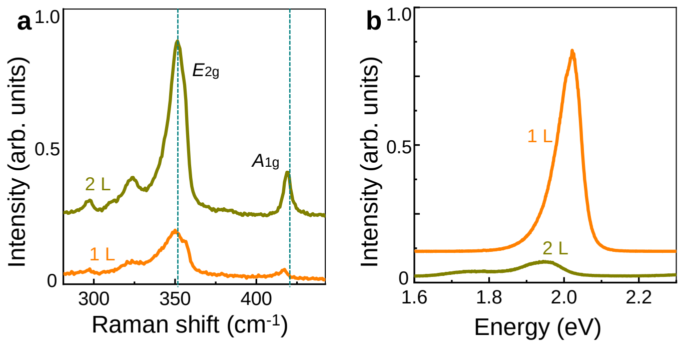

Supplementary Fig. 3 | Raman (a) and PL (b) spectra of the monolayer and bilayer WS_2 samples.

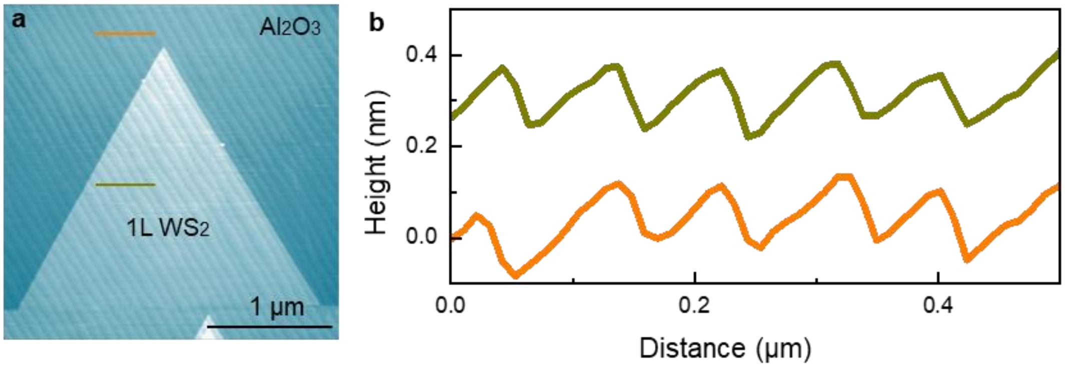

Supplementary Fig. 4 | Surface morphology of monolayer WS_2 grown on Al_2O_3 substrate. a, AFM image of a monolayer WS_2 island grown on *a*-plane sapphire. **b,** Height profile of the surface on bare sapphire and monolayer WS_2. The morphology of the steps on sapphire substrate can be perfectly replicated to the as-grown monolayer WS_2.

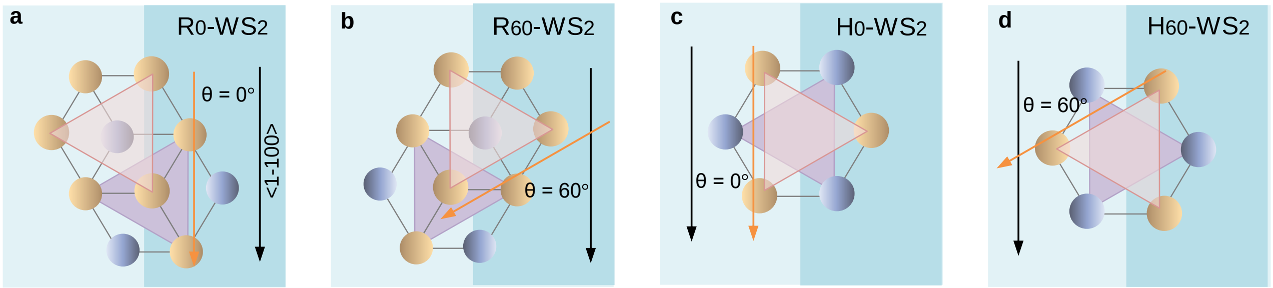

Supplementary Fig. 5 | Schematic diagrams of the definition of R_0-WS_2 (a), R_{60}-WS_2 (b), H_0-WS_2 (c), and H_{60}-WS_2 (d).

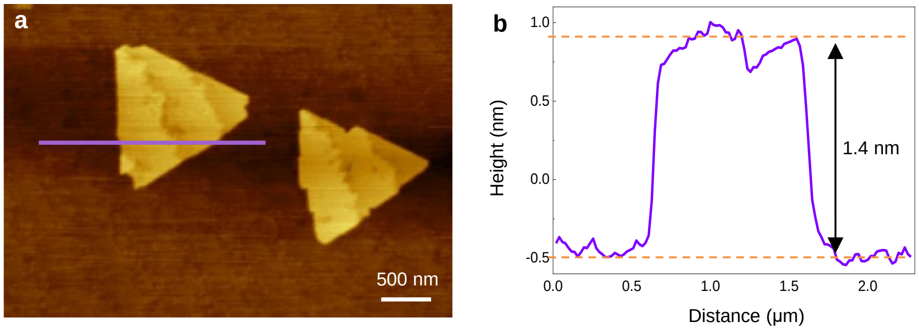

Supplementary Fig. 6 | AFM measurement of bilayer WS_2 islands. a, AFM image of two WS_2 islands at early growth stage. **b,** Height profile of **(a)**, indicating the bilayer WS_2 nucleation.

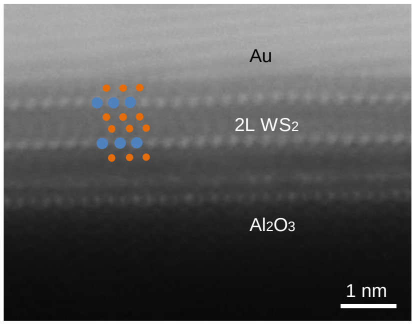

Supplementary Fig. 7 | Cross-sectional TEM image of bilayer WS_2 samples.

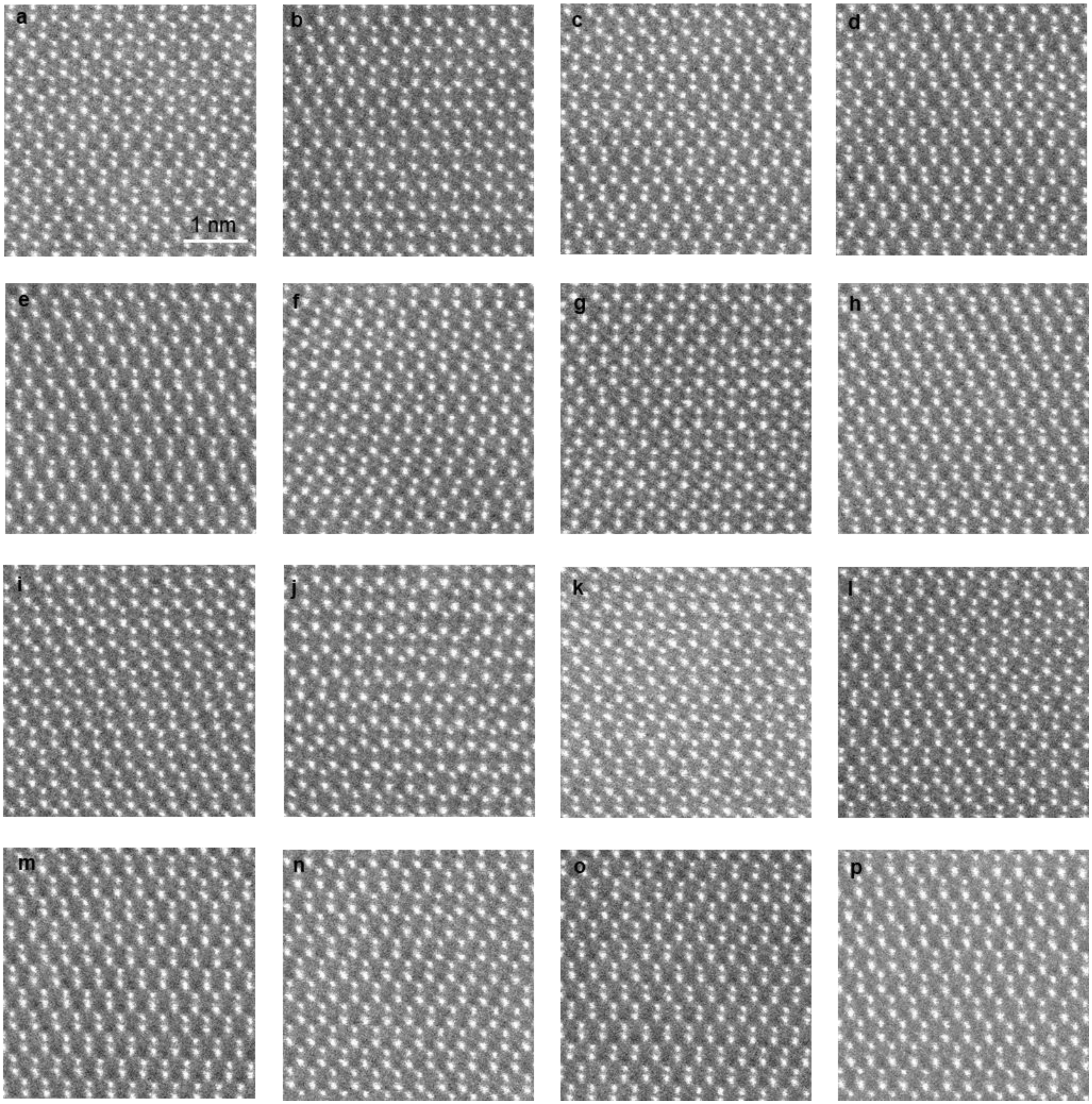

Supplementary Fig. 8 | a-p, Atomically-resolved TEM images of the bilayer WS_2, the lattice orientations of WS_2 at different areas are nearly the same. The image sizes of **(a-p)** are the same.

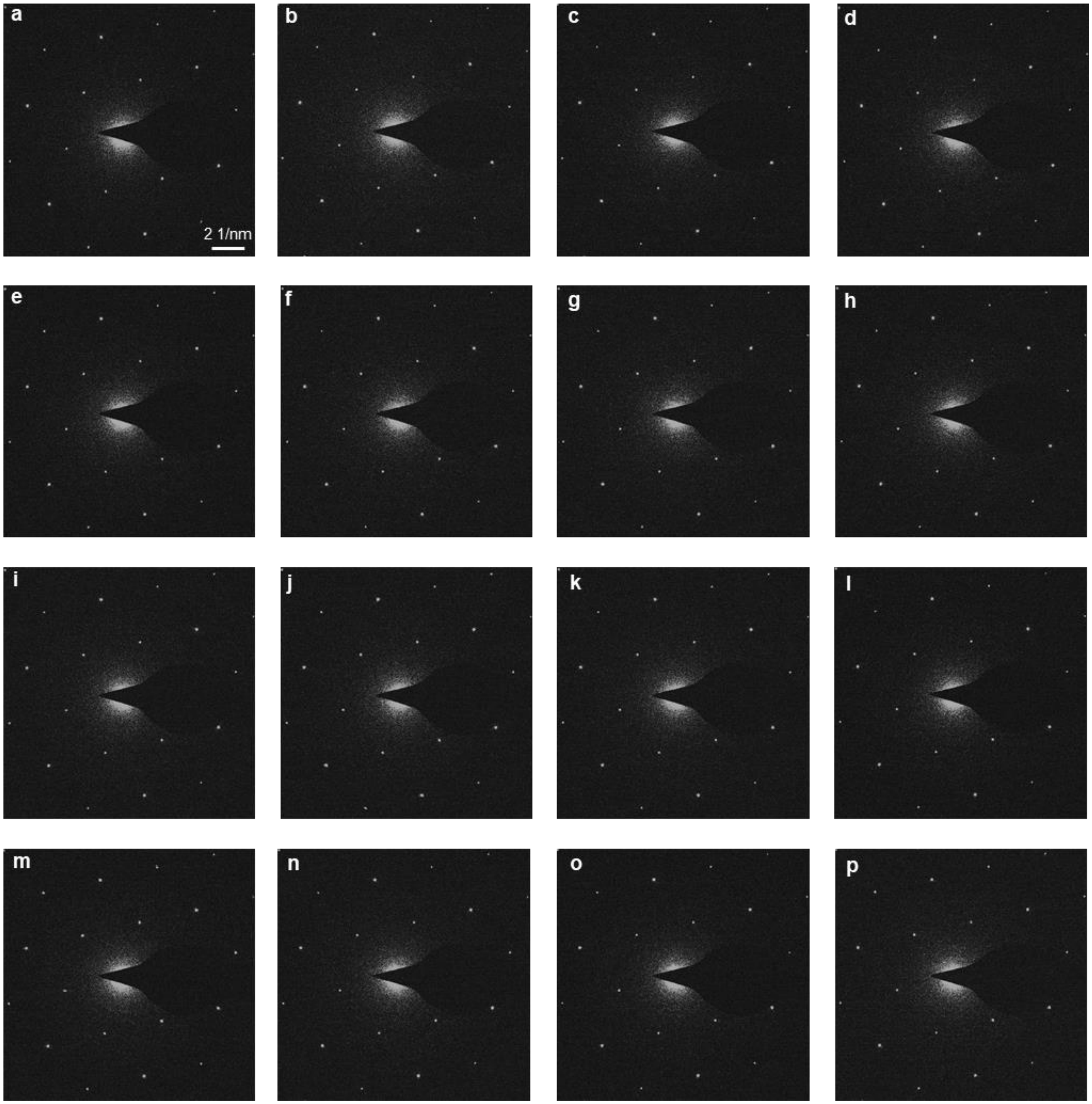

Supplementary Fig. 9 | a-p, SAED patterns of the bilayer WS_2 samples at different areas of the sample, demonstrating nearly identical orientations. The image sizes of **(a-p)** are the same.

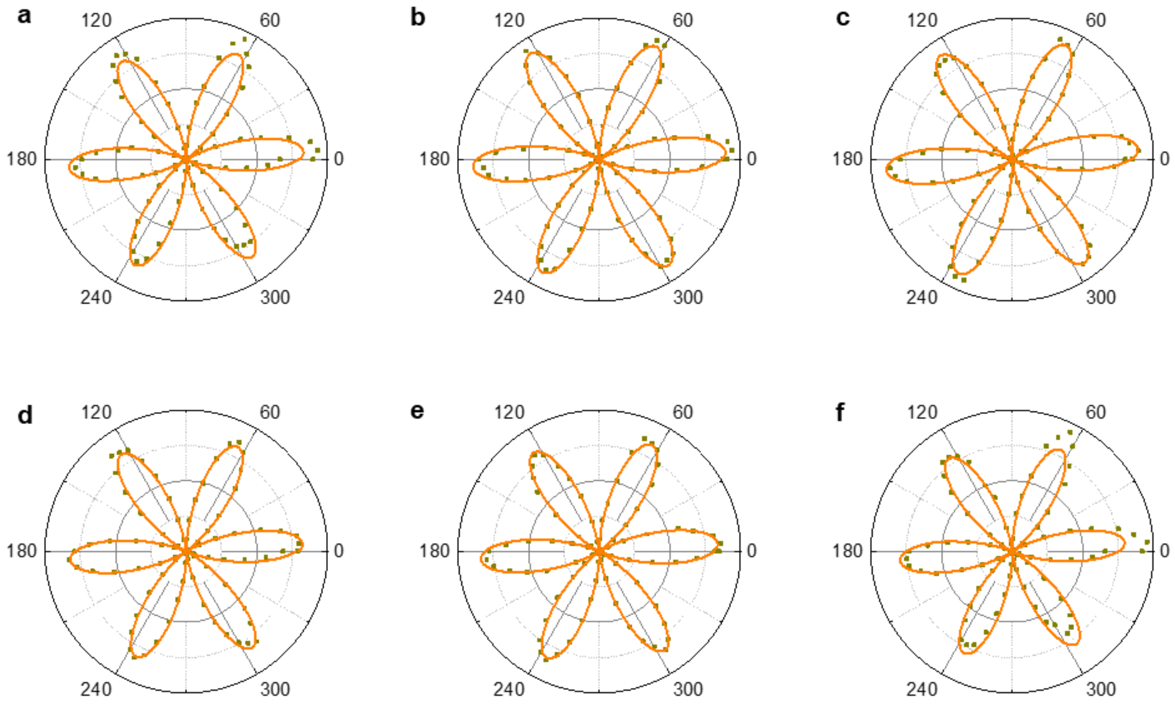

Supplementary Fig. 10 | Single-crystal nature of bilayer WS_2 samples. a-f, Representative linearly polarized SHG six-petal patterns of bilayer WS_2 at different positions. The nearly same polarization-dependent SHG patterns confirm the single crystallinity.

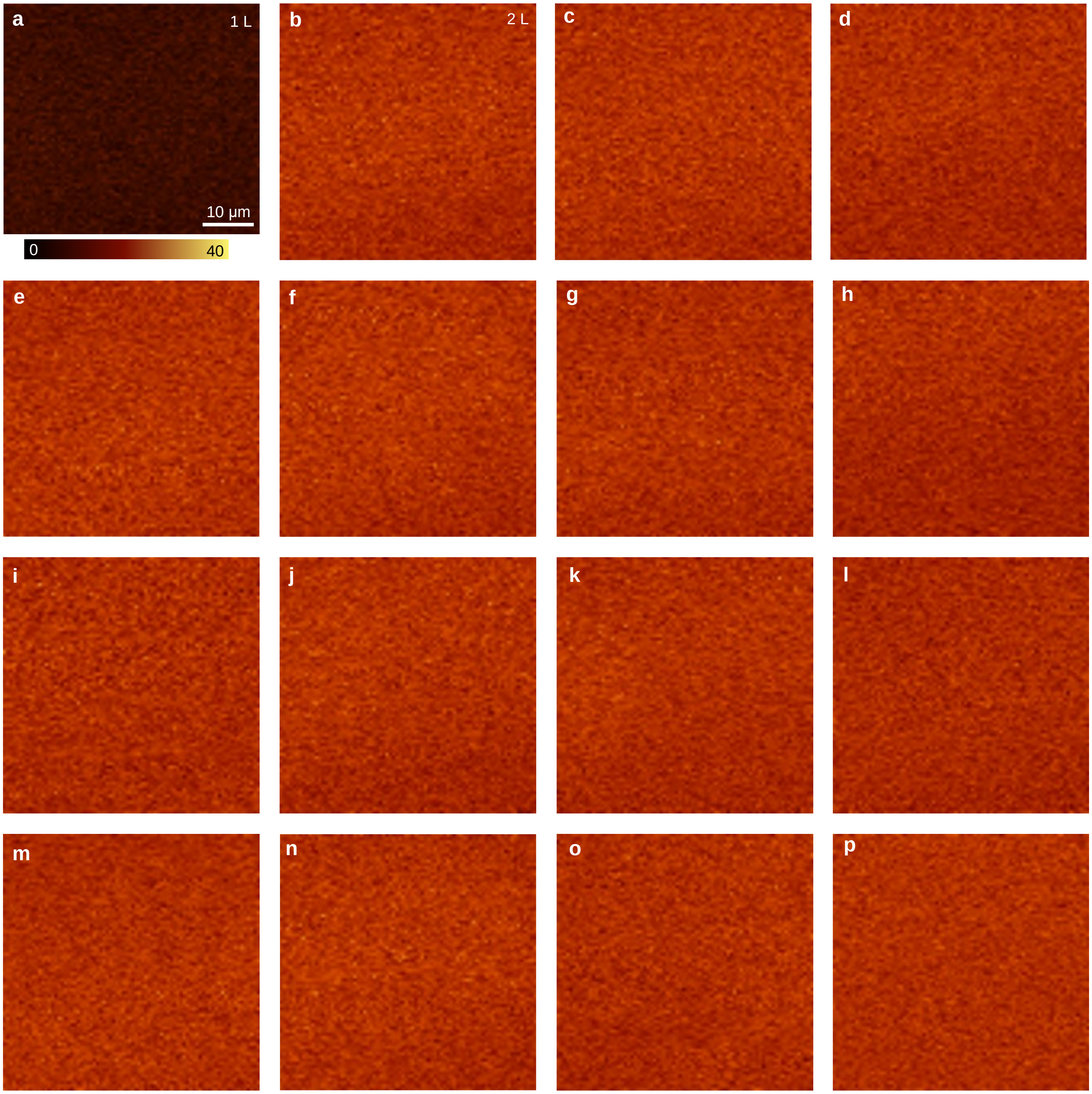

Supplementary Fig. 11 | a, SHG mapping of a monolayer WS_2 sample. b-p, SHG mapping of bilayer WS_2 samples at different areas. No dark lines can be observed, demonstrating the seamless stitching of different WS_2 islands. The image widths of **(a-p)** are the same.

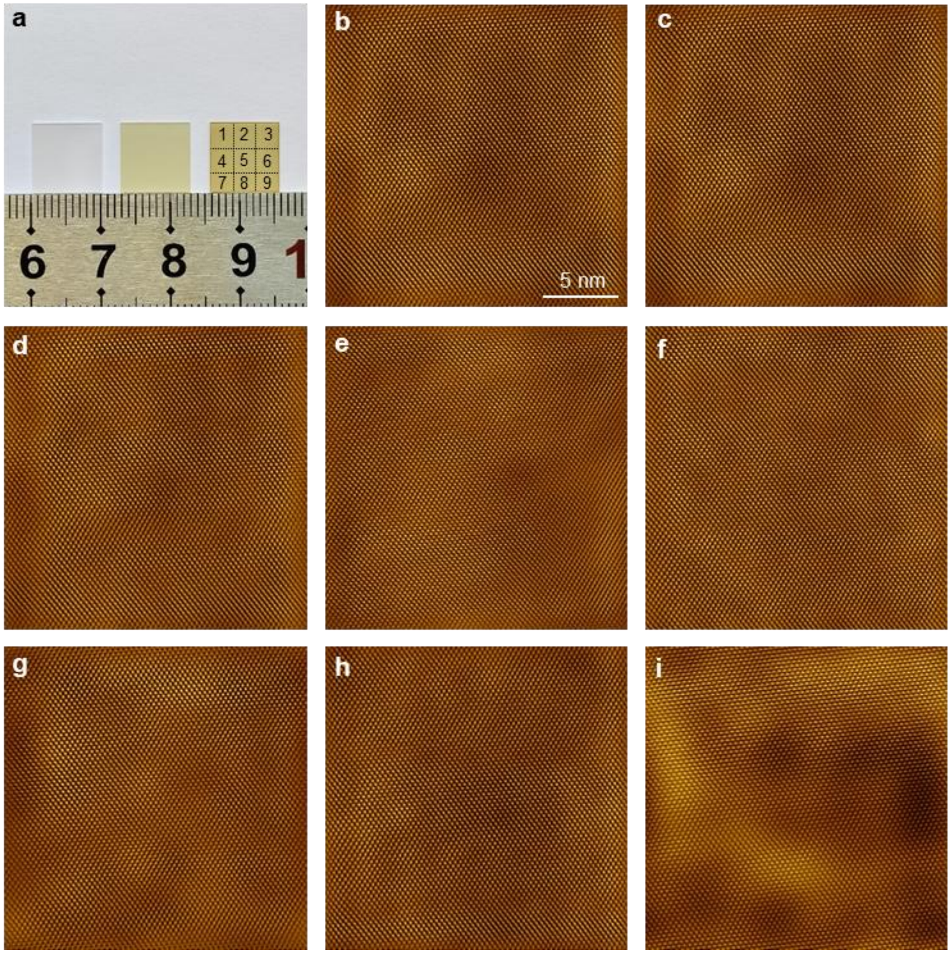

Supplementary Fig. 12 | AFM characterizations of bilayer WS_2 samples. a, Optical image of the bilayer WS_2 sample, the AFM characterizations were conducted at different marked areas. **b-i,** Atomically-resolved AFM images of the bilayer WS_2, the lattice orientations of WS_2 at different areas are nearly the same. The image sizes of **(b-i)** are the same.

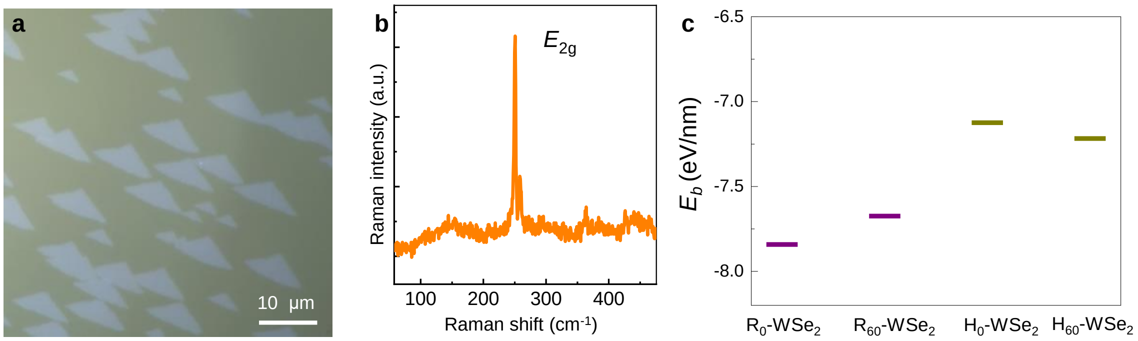

Supplementary Fig. 13 | a, Optical image of aligned bilayer WSe_2 islands. **b,** Raman spectrum of bilayer WSe_2. **c,** Binding energies of R- and H-stacked bilayer WSe_2 that across an atomic step on a-plane sapphire.

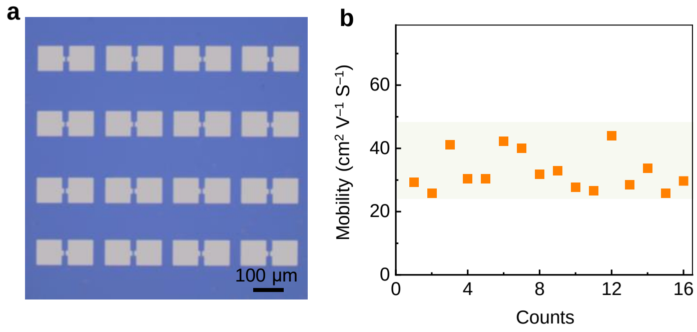

Supplementary Fig. 14 | a, Optical image of a 4×4 device array. **b,** Mobility of devices shown in **(a)** obtained at room temperature.

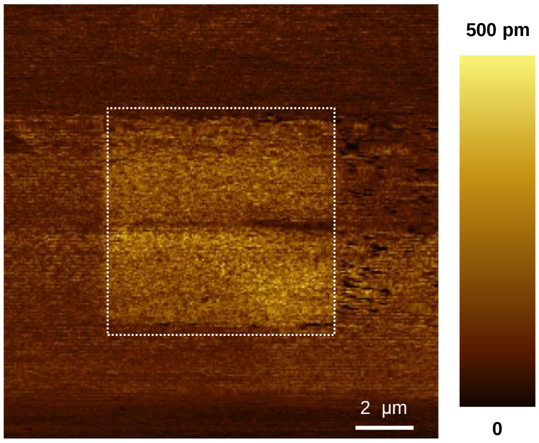

Supplementary Fig. 15 | The amplitude image of bilayer WS_2 recorded after polarization switching with DC bias +4 V in the central region.

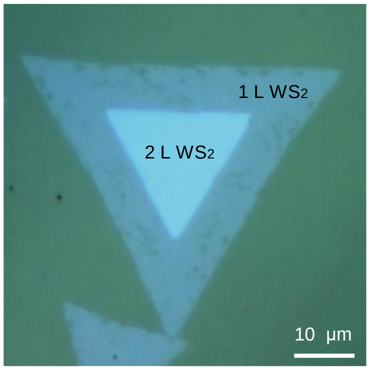

Supplementary Fig. 16 | Optical image of an R-stacked bilayer WS_2 island with monolayer areas after being exposed to air for two months.
